# Supplementary material for: A high-resolution mRNA expression time course of embryonic development in zebrafish
Source: eLife. 2017 Nov 16;6:e30860. doi: 10.7554/eLife.30860 (PMC5690287; doi:10.7554/eLife.30860)
Supplement: Supplementary file 6. [file elife-30860-supp6.zip › biolayout-clusters-files/Cluster127.html]

Cluster127


# Cluster127: Detail

### Go to ZFA detail

## GO

| GO ID | Description | Domain | Annotated | Expected | Observed | Adjusted p-value | Genes | Ensembl IDs |
| --- | --- | --- | --- | --- | --- | --- | --- | --- |
| GO:0035097 | histone methyltransferase complex | cellular\_component | 17 | 0.01 | 2 | 0.0145 | ruvbl1 rbbp5 | ENSDARG00000002591 ENSDARG00000042147 |
| GO:0004003 | ATP-dependent DNA helicase activity | molecular\_function | 14 | 0.01 | 2 | 0.0086 | ruvbl1 blm | ENSDARG00000002591 ENSDARG00000077089 |
